# Editorial: The immunological regulation of extracellular vesicles on chronic diseases

**DOI:** 10.3389/fimmu.2024.1442387

**Published:** 2024-06-18

**Authors:** Renwen Wan, Peng Chen, Shicheng Guo, Jinhong Zhu, Jie Mei, Chun Wai Mai, Zhiwen Luo

**Affiliations:** ^1^ Department of Sports Medicine, Huashan Hospital, Fudan University, Shanghai, China; ^2^ Department of Sports Medicine, Peking University Shenzhen Hospital, Shenzhen, China; ^3^ Department of Medical Genetics, University of Wisconsin (UW)-Madison, Madison, WI, United States; ^4^ Department of Laboratory Medicine, Biobank Harbin Medical University Cancer Hospital, Harbin, Heilongjiang, China; ^5^ The First Clinical Medicine College, Nanjing Medical University, Nanjing, China; ^6^ Department of Pharmaceutical Chemistry, Faculty of Pharmaceutical Sciences, University College Sedaya International (UCSI), Kuala Lumpur, Malaysia

**Keywords:** extracellular vesicles (EVs), chronic diseases, immunology, mechanisms, biomarker

Extracellular vesicles (EVs) are microscopic membrane structures that originate inside the cell and are then expelled into the extracellular matrix, especially exosomes (1). EVs are encapsulated by a lipid bilayer and harbor a variety of biomolecules, including proteins, lipids, and various forms of RNA and DNA. Primarily, EVs have been considered cellular waste. However, most researchers now found that EVs play a key role in mediating complex cellular communication (2–4). After over 30 years of exploration, the regulation of exosomes in intercellular transport mechanisms was further explored in depth. Scientists James E. Rothman, Randy W. Schekman, and Thomas C. Südho were jointly awarded the 2013 Nobel Prize for their outstanding contributions to this field. The cellular interactions responsible for exosomes are critical for a myriad of physiological processes and have implications for the pathogenesis of disease (5, 6). As our understanding of EVs continues to grow, the field has undergone a major shift and has begun to explore the potential of EVs for diagnostic and therapeutic applications (7–10). This collection of manuscripts on our topic - The Immunological Regulation of Extracellular Vesicles on Chronic Diseases, provides a comprehensive overview of the latest advances in EVs and immunology research. Experts have written 11 featuring articles in their respective fields. This Research Topic not only reveals innovative approaches to isolate and characterize EVs but also explores the functional roles of EVs in the regulation of chronic diseases. In addition, this Research Topic demonstrated EVs’ emerging applications in the fields of targeted therapies and biomarker discovery shown in **Figure 1**.

**Figure 1:**
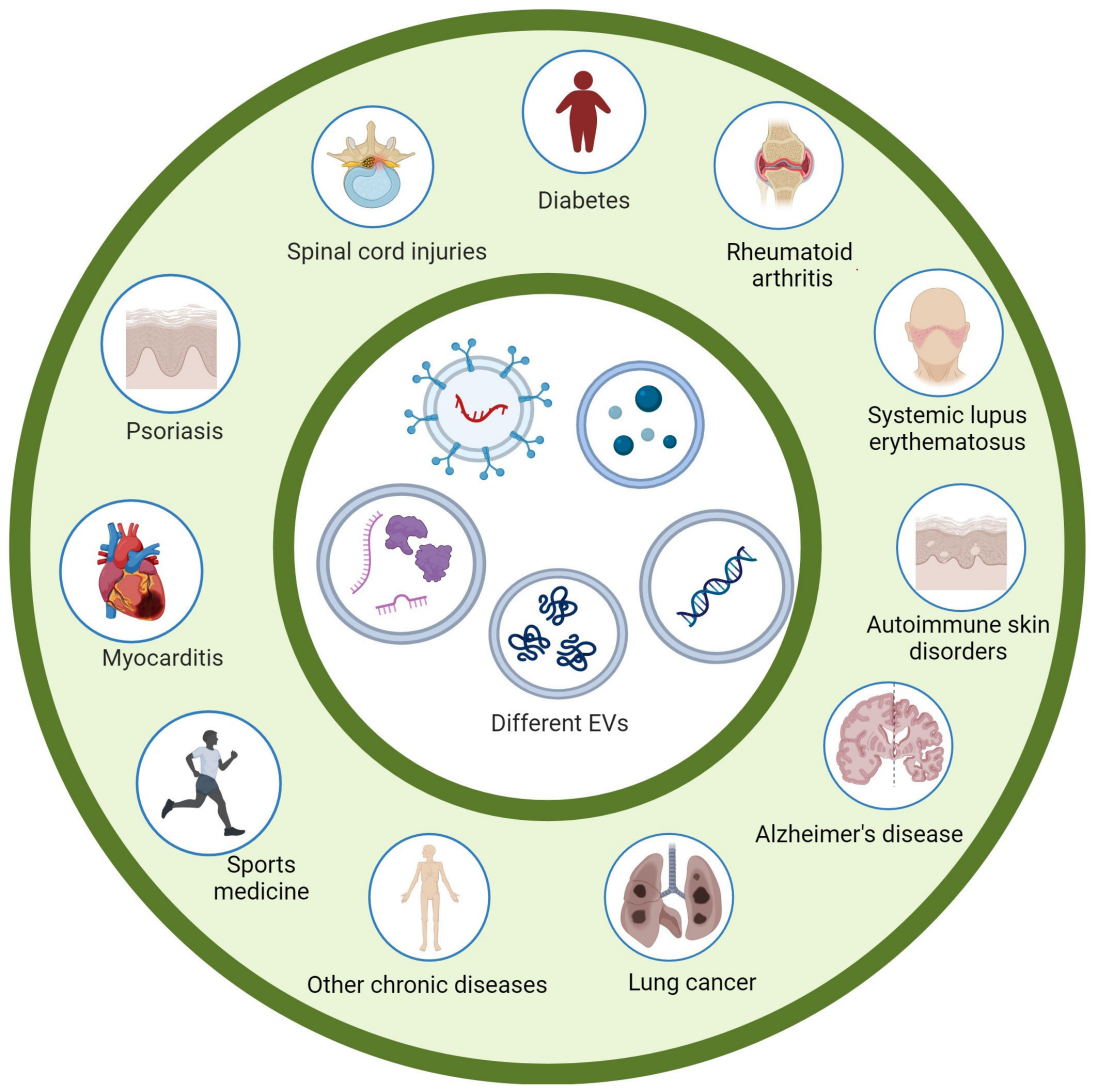
Various extracellular vesicles can play an immunomodulatory role in many chronic diseases.

An innovative study conducted by Lentilhas-Graca et al. investigated the impact of macrophage secretomes on recovery from spinal cord injuries. The research revealed that macrophage secretomes, exhibiting diverse polarization patterns, exert varied influences on neuronal growth and survival. Notably, secretomes activated by IL-10 and TGF-β1 were found to significantly enhance axonal regeneration and contribute to functional recovery post-spinal cord injury. Proteomic analysis identified a suite of proteins within these secretomes that are pivotal in axon extension and the establishment of cell polarity, offering novel therapeutic avenues for spinal cord injury treatment.

In a comprehensive review, Iuliano et al. delineate the involvement of EVs in the pathogenesis of psoriasis and their potential therapeutic applications. The review articulates the critical function of EVs as conveyors of molecular signals across the psoriatic landscape, their utility as innovative biomarkers, and their capability as platforms for precision anti-inflammatory treatments. Furthermore, the discussion extends to the integration of EVs within the psoriasis microenvironment, their role in disease transmission, and the progression of related comorbidities, underscoring the potential of EV-based biotechnologies in both therapeutic and research settings.

Di Florio et al.’s review focuses on the role of mitochondrial EVs in autoimmune diseases, particularly myocarditis. The review highlights that viruses like Coxsackievirus B3 and SARS-CoV-2 can induce cells to release mitochondrial vesicles during infection, which subsequently trigger an immune response culminating in autoimmune reactions. Moreover, the presence of mitochondrial autoantibodies in myocarditis patients and the regulatory role of autoimmune regulatory factors (AIRE) in mitigating mitochondrial antigen-induced autoimmunity are explored. This study offers fresh perspectives on the mechanisms through which viral infections may precipitate autoimmune conditions.

A review conducted by Zhang et al. provides a comprehensive analysis of the roles of exosomes derived from various cellular origins in the context of rheumatoid arthritis (RA). The study elucidates that exosomes are intricately involved in the pathogenesis of RA and may serve pivotal roles as diagnostic markers and therapeutic agents. Notably, exosomes originating from mesenchymal stem cells demonstrate considerable potential in modulating immune responses, mitigating inflammation, and facilitating tissue repair, suggesting their viability as therapeutic modalities in RA management.

In a succinct mini-review, Zhang et al. highlight the critical functions of exosomal microRNAs (miRNAs) in autoimmune skin disorders. This review details how these miRNAs, abnormally expressed across various autoimmune skin conditions, influence disease progression by regulating the secretion of essential cytokines and directing immune cell differentiation. The potential of exosomal miRNAs as biomarkers for tracking disease activity, recurrence, and therapeutic response is underscored, paving the way for novel targeted treatment approaches. The review calls for further investigation into the mechanisms of exosomal miRNAs to enhance clinical treatment strategies.

Ye et al.’s mini-review discusses the immunomodulatory properties of mesenchymal stem cell-derived extracellular vesicles (MSC-EVs) in Alzheimer’s disease (AD). The review proposes MSC-EVs as innovative agents for AD therapy by detailing their roles in suppressing glial cell activation, reducing inflammatory cytokine levels, and promoting both neuroprotection and amyloid β clearance. It also critically assesses the potential challenges and advantages of MSC-EVs in clinical applications, offering valuable insights for advancing extracellular vesicle-based therapies for neurodegenerative diseases.

Zhao et al.’s mini-review examines the involvement of exosomes in lung cancer progression, focusing on their roles in metastasis, diagnostic potential, and immunological interactions. Exosomes are described as crucial players in lung cancer dynamics, capable of enhancing metastatic processes and modulating immune responses. The review highlights the diagnostic potential of specific miRNAs within exosomes and discusses the innovative applications of engineered exosomes in lung cancer therapy. It also emphasizes the need for further studies to validate the safety and efficacy of exosomal applications in clinical settings.

A review authored by He et al. critically assesses the immunomodulatory functions and therapeutic potentials of natural killer cell-derived extracellular vesicles (NKEVs) in managing chronic diseases. These vesicles are enriched with a diverse array of cytotoxic proteins and nucleic acids, demonstrating promising therapeutic effects across various conditions, including malignant tumors, hepatic fibrosis, and pulmonary injuries. Despite certain challenges such as limited yield and suboptimal targeting capabilities, advancements in research concerning memory-like NK cells, their derived EVs, and engineered NKEVs are paving the way for enhanced treatment efficiency, specificity, and safety. Collectively, NKEVs are emerging as potent therapeutic agents in the realm of chronic disease management.

In the mini-review by Huang et al., the role of exosomes in sports medicine is explored, emphasizing their importance in managing chronic conditions and boosting athletic performance. The review elucidates the fundamental aspects of exosomes, including their biogenesis, release mechanisms, content profiles, and biological activities, and discusses their capabilities in facilitating muscle repair, arthritis treatment, and performance enhancement. The paper also addresses the ongoing challenges and future prospects of exosome application in sports medicine, underscoring their significant role in personalizing treatment and advancing clinical evaluations and technological innovations.

Wong et al. provide a comprehensive review on the use of mesenchymal stem cell (MSC)-derived extracellular vesicles (MSC-EVs) in treating systemic lupus erythematosus (SLE). The review highlights the vast potential of MSC-EVs as innovative, cell-free therapeutic options that leverage immunomodulation, MSC preconditioning techniques, and their diagnostic and therapeutic applications in SLE. It also points to existing gaps in understanding the precise mechanisms of MSC-EV actions and the hurdles in their clinical implementations, advocating for more research to optimize their therapeutic deployment in SLE.

Li et al.’s review offers an in-depth look at the emerging role of exosomes in the immunotherapy of diabetes. Serving as critical intercellular communicators, exosomes can reprogram immune responses associated with diabetes and its complications. This paper discusses how exosomes from immune cells like neutrophils, T lymphocytes, and macrophages, as well as from stem cells, exert immunomodulatory and anti-inflammatory effects in diabetes management. The review also considers engineered exosomes as novel therapeutic tools for diabetes, addressing current challenges in their clinical application and proposing new directions for future diabetes immunotherapy research.

Through these articles, we have not only expanded our understanding of the function of EVs but also opened new perspectives for future therapeutic strategies. This album is the result of our joint efforts and demonstrates how scientific research can reveal deeper mechanisms in biology and bring hope for the treatment of chronic diseases. We look forward to continuing this exciting journey of scientific discovery of EVs with researchers around the world.

## Author contributions

RW: Writing – review & editing, Writing – original draft. PC: Writing – review & editing, Writing – original draft. SG: Writing – review & editing, Writing – original draft. JZ: Writing – review & editing, Writing – original draft. JM: Writing – review & editing, Writing – original draft. CM: Writing – review & editing, Writing – original draft. ZL: Writing – review & editing, Writing – original draft, Visualization, Validation, Supervision, Software, Resources, Project administration, Methodology, Investigation, Funding acquisition, Formal analysis, Data curation, Conceptualization.
